# Electrostimulation of the carotid sinus nerve in mice attenuates inflammation via glucocorticoid receptor on myeloid immune cells

**DOI:** 10.1186/s12974-020-02016-8

**Published:** 2020-12-02

**Authors:** Aidan Falvey, Fabrice Duprat, Thomas Simon, Sandrine Hugues-Ascery, Silvia V. Conde, Nicolas Glaichenhaus, Philippe Blancou

**Affiliations:** 1 Université Côte d’Azur, CNRS, Institut de Pharmacologie Moléculaire et Cellulaire, Valbonne, France; 2 E-Phy-Science, Valbonne, France; 3 CEDOC, NOVA Medical School, Faculdade de Ciências Médicas, Universidade NOVA de Lisboa, Lisboa, Portugal

**Keywords:** Bioelectronic medicine, Carotid body, Carotid sinus nerve, Corticosterone, Electrostimulation, Immunology

## Abstract

**Background:**

The carotid bodies and baroreceptors are sensors capable of detecting various physiological parameters that signal to the brain via the afferent carotid sinus nerve for physiological adjustment by efferent pathways. Because receptors for inflammatory mediators are expressed by these sensors, we and others have hypothesised they could detect changes in pro-inflammatory cytokine blood levels and eventually trigger an anti-inflammatory reflex.

**Methods:**

To test this hypothesis, we surgically isolated the carotid sinus nerve and implanted an electrode, which could deliver an electrical stimulation package prior and following a lipopolysaccharide injection. Subsequently, 90 min later, blood was extracted, and cytokine levels were analysed.

**Results:**

Here, we found that carotid sinus nerve electrical stimulation inhibited lipopolysaccharide-induced tumour necrosis factor production in both anaesthetised and non-anaesthetised conscious mice. The anti-inflammatory effect of carotid sinus nerve electrical stimulation was so potent that it protected conscious mice from endotoxaemic shock-induced death. In contrast to the mechanisms underlying the well-described vagal anti-inflammatory reflex, this phenomenon does not depend on signalling through the autonomic nervous system. Rather, the inhibition of lipopolysaccharide-induced tumour necrosis factor production by carotid sinus nerve electrical stimulation is abolished by surgical removal of the adrenal glands, by treatment with the glucocorticoid receptor antagonist mifepristone or by genetic inactivation of the glucocorticoid gene in myeloid cells. Further, carotid sinus nerve electrical stimulation increases the spontaneous discharge activity of the hypothalamic paraventricular nucleus leading to enhanced production of corticosterone.

**Conclusion:**

Carotid sinus nerve electrostimulation attenuates inflammation and protects against lipopolysaccharide-induced endotoxaemic shock via increased corticosterone acting on the glucocorticoid receptor of myeloid immune cells. These results provide a rationale for the use of carotid sinus nerve electrostimulation as a therapeutic approach for immune-mediated inflammatory diseases.

## Introduction

Inflammation is part of the complex biological response of body tissues to harmful stimuli—pathogens, damaged cells or irritants. It involves the recruitment of immune cells and the production of soluble molecules including pro-inflammatory cytokines—tumour necrosis factor (TNF), interleukin (IL)-1α, IL-1β, IL-6 and IL-12. These cytokines eventually act on both immune and non-immune cell types by signalling through specific surface receptors. While inflammation could be viewed as a protective mechanism, pro-inflammatory cytokines may also cause tissue injury and have a deleterious effect. This occurs during endotoxic shock which results from a severe, generalised inflammatory response induced by bloodstream infection with gram-negative bacteria. It also occurs in immune-mediated inflammatory diseases (IMIDs) such as rheumatoid arthritis (RA), inflammatory bowel disease (IBD) and systemic lupus erythematosus (SLE) [1]. It is therefore not surprising that several neuro-hormonal anti-inflammatory pathways have been identified. At least two neuro-hormonal anti-inflammatory pathways have been described: the activation of the hypothalamic-pituitary-adrenal (HPA) axis and the vagal anti-inflammatory reflex [2].

The HPA is activated by several stimuli including psychological stress, which activate the paraventricular nucleus (PVN) of the hypothalamus and eventually the release of cortisol-releasing hormone (CRH) into the anterior pituitary. In turn, CRH induces the release of adrenocorticotrophic hormone (ACTH) into the blood which stimulates the production of glucocorticoids by the cortex of the adrenal glands. Glucocorticoids are potent anti-inflammatory molecules, and their effect is mediated via signalling by the glucocorticoid receptor (GR) which is expressed by almost all cells in the body and in particular innate immune cells [3].

While the inhibition of pro-inflammatory cytokine production by immune cells is mediated by glucocorticoids when the HPA axis is activated, the vagal anti-inflammatory reflex relies on the binding of acetylcholine (ACh) on nicotinic ACh receptors (nAChR). The vagal anti-inflammatory reflex primarily involves the vagus nerve, synapsing at the coeliac ganglion, and the release of norepinephrine by sympathetic nerve fibres that project to the spleen. Norepinephrine binds to β2 adrenergic receptor (AR) at the surface of CD4^+^ T cells, eventually inducing the release of ACh and the inhibition of pro-inflammatory cytokine production by spleen macrophages through a nAChR-dependent mechanism [4]. In recent years, efforts to convert this inflammatory reflex into a therapeutic have been conducted via electrical activation of the vagus nerve. This form of therapeutic can be described as bioelectronic medicine, and clinical trials have been performed to investigate its anti-inflammatory properties in IMIDs [5, 6]. Partially, due to the studies on the inflammatory reflex, bioelectronic medicine has seen a resurgence in recent years [7]. There is a lot of interest in discovering the anti-inflammatory potential of additional nerves. One interesting target is potentially the carotid sinus nerve (CSN).

The CSN is connected to the carotid body (CB) and baroreceptors that project to the brain [8]. The CB is a paraganglion located bilaterally in the neck at the bifurcation of the carotid artery into the internal and external arteries. It is a polymodal sensor and is capable of detecting oxygen and carbon dioxide concentration in the blood and insulin [8, 9]. Baroreceptors are sensors located in the carotid sinus and in the aortic arch, which sense the blood pressure and relay the information to the brain via the CSN or the aortic depressor nerve, respectively. Once stimuli are detected by the CB or baroreceptors, they signal via the CSN to the brain to modulate these physiological variables as required. In recent years, it is becoming increasingly evident that both the CB and the baroreceptors can detect inflammation. For example, the CB can detect cytokines—TNF, IL-1B and IL-6 [10–12]—and pathogenic components—lipopolysaccharide (LPS) and zymosan [11, 13, 14]. This detection causes activation of the CB [10, 12], as well as inducing signalling in the CSN [11, 15]. Additionally, baroreceptors also express receptors for inflammatory mediators, and stimulation of immune receptors with their cognate ligands would lead to activation of C-fibre neurons [16]. Most importantly, there is preliminary evidence suggesting that both the CB [17] and the baroreceptors [18] signalling to the brain could be anti-inflammatory in rats. In line with these results, it was found that bilateral removal of the CSN in rats reduced survival when these animals are exposed to high bacterial load [19]. Overall, the evidence suggests that selective activation of the CSN may attenuate inflammation. Therefore, we hypothesised that electrical activation of the CSN in mice will attenuate inflammation and protect against IMIDs.

## Methods

### Animals

Female 6–7-week-old C57BL/6 mice were purchased from Charles River, France. All experiments were conducted on mice age 8–10 weeks old. LysM-Cre:GR^fl/fl^ mice were backcrossed more than 12 times onto the C57BL/6 background and confirmed to be double-positive by genomic PCR (GoTaq Green Master Mix, Promega). All mice were given access to food and water ad libitum and maintained on a 12-h light and dark schedule.

### Reagents

Lipopolysaccharide (LPS) from *Escherichia coli* (O127:B8) (Sigma-Aldrich) was aliquoted to 5 mg/ml. The aliquots were frozen at − 20 °C and defrosted to be prepared fresh with PBS to a concentration of 100 μg in 200 μl intraperitoneally (IP). Prior to surgery, buprecare (Axience, Centravet, France) was injected at 0.2 mg/kg IP, and the following day, mice were injected with an additional 50 μl subcutaneously. Dolethal (Vétoquinol, Centravet, France) was used to induce lethal unconsciousness prior to intracardial blood extraction. Atropine, hexamethonium, propranolol and mifepristone (all from Sigma-Aldrich) were used at a concentration of 1 mg/kg, 10 mg/kg, 2.5 mg/kg and 80 mg/kg, respectively. All were diluted in PBS except for mifepristone which was diluted in a PBS/DMSO mix (10%) (Sigma-Aldrich). All were injected IP in 200 μl.

### Acute CSN stimulation

Mice were injected IP with buprecare (100 μl—Axience, Centravet, France), as an analgesic, at least 10 min prior to surgery. Isoflurane (Piramal, CSP, France) mixed with air at 2.5% was used to induce mice into unconsciousness. The left carotid artery bifurcation was exposed, and the hypoglossal nerve was located. A small muscle covering the hypoglossal nerve was located and cut solely. The CSN was located via its connection to the CB.

**Fig. 1:**
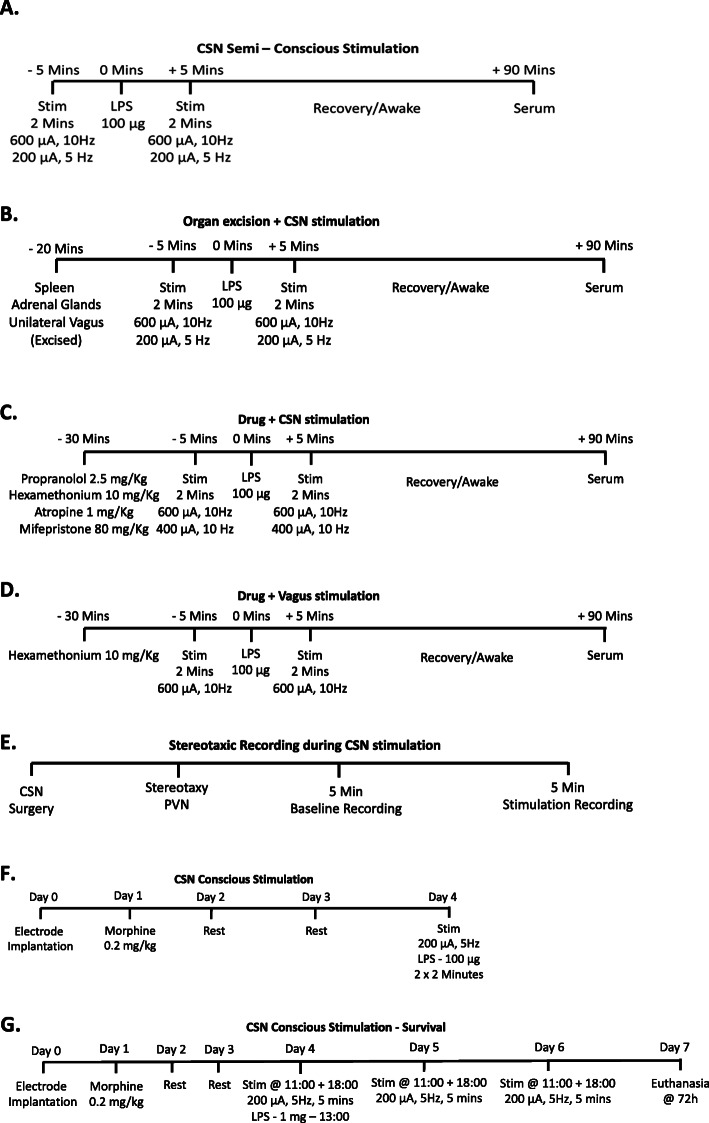
Timelines for experimental protocols. Eight to 10-week-old C57BL/6 mice were used in various experimental protocols. Prior to CSN surgery, some experiments had organ/nerve excisions (**b**), and in others, blocking drugs were administered (**c**, **d**). In all instances with recovery, mice recovered in a heated cage (**a**–**d**, **f**, **g**). LPS was administered IP (200 μl) in all instances at 100 μg (**a**–**d**, **f**, except **g**) when a lethal dose of LPS was used (1 mg–200 μl). When stimulation was required (**a**–**g**), it was administered at 600 μA + 10 Hz, 400 μA + 10 Hz or 200 μA + 5 Hz and 0.1 ms in all instances. Stimulation occurred during unconsciousness (**a**–**e**) and consciousness (**f**, **g**). Experiments were typically conducted at least twice with an *n* number of 6–8

In sham and experimental mice, home-made nichrome electrodes made of two individual wires (A&M Systems) were placed under and around the left CSN. Experimental mice only received electrostimulation, and sham mice did not. For all acute experiments, electrical stimulation was administered by a Plexon stimulator (PlexStim Electrical Stimulator System) as rectangular charged-balanced biphasic pulses with 200 to 600 μA pulse amplitude, 100 μs pulse width (positive and negative) at 5 or 10 Hz frequency for 2 min, 5 min before and after a 100-μg LPS (Sigma-Aldrich) IP injection in 200 μl of PBS (Fig. 1a–d). Organ excision and drug injection were performed 20 and 30 min prior to LPS injection, respectively (Fig. 1b–d). As previously observed by us and others, LPS-induced serum TNF levels vary from one experiment to another due to the time of the day it was injected or the LPS lot-to-lot variations. A variety of stimulation patterns were shown to be effective; however, depending on the manufactured electrodes, the optimal stimuli varied. Typically, the lowest impact stimulation pattern that was effective was chosen to decrease the likelihood of electrical spread to other nerves. Exact stimulation patterns are outlined per experiment (Fig. 1). Once stimulation was complete, mice were sutured and allowed to recover in a heated cage. Blood was collected 90 min after LPS injection retro-orbitally just before being sacrificed.

### Chronic CSN stimulation

Surgery was performed as described for acute CSN stimulation. An additional step was added to enable further anchoring of the physiological glue (Kwik-cast & Kwik-sil, World Precision Instruments) to ensure the wires were kept safely in place. The animal was cervically sutured and gently flipped to place dental cement (Super Bond C&B) around the electrode end to build the head cap, prior to placing the mouse in a heated cage for recovery. The following day, mice were given 4 μg of morphine (Burprecare) subcutaneously, and the subsequent 2 days were undisturbed. Mice were placed into individual cages, and a stimulating wire was connected to their head caps when needed.

Inhibition of LPS-induced TNF release was assessed following timeline F (Fig. 1f) in two independent cohorts. Mice were electrically stimulated and injected with LPS as described above. Ninety minutes after LPS injection, mice were given Dolethal (Vétoquinol, Centravet, France) before blood was extracted intracardially.

Survival to lethal LPS injection was assessed following IP injection of 1 mg of LPS. Conscious stimulation was performed as described, following timeline G (Fig. 1g). For this experiment, however, on the first day, mice were stimulated (200 μA, 5 Hz, 0.1 ms) for 5 min at 11:00 and again at 18:00. LPS was injected at 13:00, the mice were watched for the next 72 h and when a mouse died the hour was noted.

### Organs excision and vagus nerve resection

The spleen excision was performed before electrode implantation by tying a suture knot onto the three major blood vessels entering the spleen and then gently separating out the spleen. In a separate experiment, the entire adrenal glands, bilaterally, were solely cauterised by a biological cauterising tool. In another separate experiment, prior to CSN stimulation, the left cervical vagus nerve in the vicinity of the carotid bifurcation was excised unilaterally. All mice were adequately sutured as required.

### Arterial blood pressure measurement

Mice were anaesthetised by isoflurane inhalation, and their body temperature was kept constant with a temperature controller (ATC2000, World Precision Instrument). Blood pressure was measured through a pressure catheter (outside diameter of 0.61 mm) inserted into the carotid artery. Pressure was measured with a BP-100 intravascular blood pressure transducer and acquired at 100 Hz (iWorx 214); data were acquired and analysed using LabScribe2 (iWorx Systems Inc, USA). Final traces are the mean values of data after decimation to 10 Hz.

### Vagus stimulation

Similar methods as described for CSN stimulation were used, except the cervical vagus nerve was isolated and dual nichrome wires were placed underneath it. Electrical stimulation (2 min, 600 μA, 10 Hz, 0.1 ms) was applied, 5 min before and after an IP LPS (100 μg) injection. Animals were allowed to recover as described in the “Acute CSN stimulation” section. Additionally, 30 min prior to LPS injection, atropine (1 mg/kg), hexamethonium (10 mg/kg) and propranolol (2.5 mg/kg) or sham PBS was injected IP.

### Paraventricular nucleus recording

Mice were anaesthetised with ketamine (130 mg/kg, Imalgene 1000) and xylazine (10 mg/kg) injection IP. The CSN was isolated as described above, and physiological glue was used to ensure electrodes remained in place during the stereotaxic surgery. The mice were placed in a stereotaxic frame (SR-6, Narishige, Japan), and a craniotomy to target the hypothalamic PVN was performed. Stereotaxic coordinates for the PVN were determined from the Paxinos and Watson rat brain atlas (2013), and stainless-steel recording electrodes (platinum/iridium wires) were implanted into the area of the PVN—which was confirmed later by dye placement. A baseline recording of PVN activity for 5 min was recorded, and subsequently, continuous stimulation (600 μA, 10 Hz, 0.1 ms) of the CSN was started, and PVN activity was recorded for 5 min using a multichannel system (Multi Channel Systems MCS GmbH, Germany). Mice were sacrificed at the end of the experiment, the brain was removed and immediately sectioned using a vibratome to view dye placement in the sections.

### Cytokines and corticosterone assays

Routinely, a TNF and corticosterone were measured by ELISA (DY410 R&D Systems and ADI-900-097, Enzo LifeSciences) as described by the manufacturers. The Meso Scale Discovery kit (V-PLEX Plus Proinflammatory Panel1 Mouse Kit) was used to assess a wide variety of cytokines (Fig. 1).

### Statistics

Data is pooled, and individual points represent one animal; results are expressed as means ± standard deviation (SD). The unpaired *t* test was used if results were shown to have a normal distribution (Shapiro-Wilks test); if not, a Mann-Whitney test was used. When the comparison between more than two experimental groups was necessary, the one-way ANOVA with Tukey post hoc test was used if the results were shown to have a normal distribution (Shapiro-Wilks test); if not, a Kruskal-Wallis with Dunn’s post hoc test was used. In all instances, *p < 0.05, **p < 0.01, ***p < 0.001 and ****p < 0.0001.

## Results

### CSN electrostimulation inhibits LPS-induced production of pro-inflammatory cytokines

**Fig. 2:**
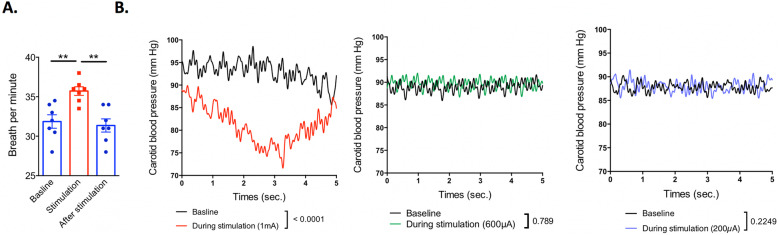
Electrostimulation of the CSN increases breath rate and decreases arterial blood pressure in mice. Eight to 10-week-old C57BL/6 mice were obtained, and CSN isolation surgery was performed. **a** Breath rate was recorded for 5 min, and the average breath per minute was calculated at baseline, during stimulation (200 μA, 5 Hz) and immediately after stimulation. Breath rate in mice before, during and after stimulation. Each individual point represents an animal, and data is expressed as means ± SD. **b** The mean carotid pressure recorded in anaesthetised mice (*n* = 3) before and during CSN electrostimulation with either 1 mA (left), 600 μA (middle) or 200 μA (right) current amplitude. Traces are averaged values

To confirm that we could activate the CSN by electrical stimulation, we placed an electrode underneath the CSN in anaesthetised mice. Applying electrical stimulation induced a transient increase in breath rate (Fig. 2a: *p* = **, one-way ANOVA, *n* = 7), therefore confirming that the electrode was correctly positioned and that it can mimic chemoreceptor activation. To test whether our chosen stimulation packages (600 μA, 10 Hz, 0.1 ms and 200 μA, 5 Hz, 0.1 ms) induced baroreceptor CSN activity, in addition to the confirmed chemoreceptor activity, we measured the arterial blood pressure from the carotid artery before, during and after stimulation (Fig. 2b, *n* = 3). A transient decrease in arterial blood pressure was recorded following 1 mA 30 Hz electrical stimulation of the CSN (*p* = ***, unpaired *t* test, *n* = 3) further confirming the location of the CSN. Interestingly, when a lower electrical stimulation was applied, 600 μA, 10 Hz or 200 μA, 5 Hz carotid blood pressure was not affected by CSN stimulation. This result suggests that we are solely inducing a chemoreceptor response from the CB/CSN.

**Fig. 3:**
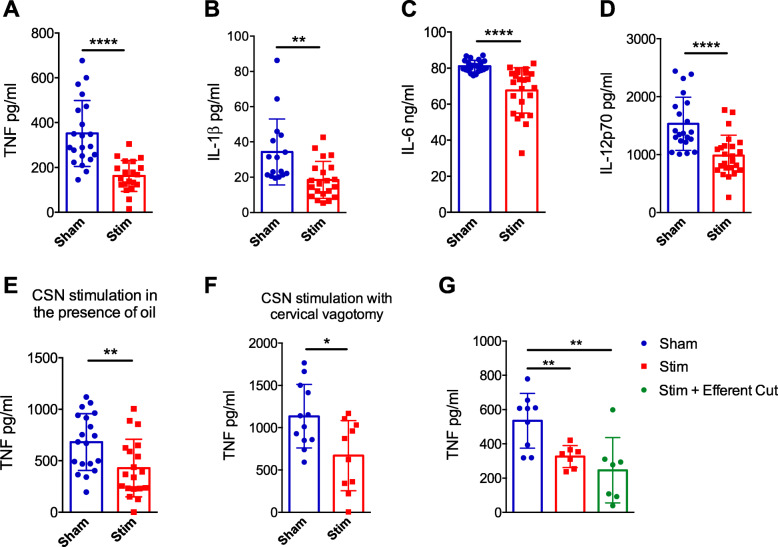
CSN electrostimulation attenuates inflammation independently of the vagus nerve. **a**–**g** Eight to 10-week-old C57BL/6 mice were anaesthetised; CSN was isolated and either cut (**g**) of left intact (**a**–**f**). Electrical stimulation was applied at 600 μA, 10 Hz (**a**–**e**) or 200 μA, 5 Hz (**f**, **g**) 5 min before and after IP LPS injection (100 μg). Blood was collected 90 min after LPS injection for serum analysis by Meso Scale Discovery (**a**–**d**) or ELISA (**e**–**g**). Impact of electrical activation of the CSN on **a** LPS-induced serum TNF levels, **b** IL-1β, **c** IL-6 and **d** IL-12p70. **e** Impact of electrical activation of the CSN on LPS-induced serum TNF levels in the presence of oil. **f** Impact of unilateral left vagal removal on LPS-induced serum TNF levels following left CSN electrostimulation. All individual points represent one animal, and data is expressed as means ± SD. **g** Impact of afferent CSN stimulation on LPS-induced serum TNF levels

To further investigate the impact of CSN electrical stimulation on LPS-induced TNF production, we injected LPS into anaesthetised mice, applied or not electrical stimulation and measured TNF serum levels 90 min later. Compared to sham-stimulated mice, CSN electrical stimulation significantly reduced the serum levels of TNF (Fig. 1a for timeline; Fig. 3a: *p* = ****; unpaired *t* test; *n* = 20–21), IL-1β (Fig. 3b: *p* = **, Mann-Whitney, *n* = 16–21), IL-6 (Fig. 3c; *p* = ****, Mann-Whitney, *n* = 22–24) and IL-12p70 (Fig. 3d; *p* = ****; Mann-Whitney, *n* = 21–24). We confirmed that these results were not due to current leakage by repeating these experiments when the CSN/electrodes were surrounded by oil (Fig. 3e; *p* = **; unpaired *t* test; *n* = 19) and upon unilateral vagus excision (Fig. 1b for timeline; Fig. 3f; *p* = *; unpaired *t* test, *n* = 10–12). We also found that the effect of CSN stimulation was mediated by an afferent signal to the brain as the impact on TNF is prevented by an efferent cut of the CSN (Fig. 3g: *p* = **, unpaired *t* test, *n* = 7–9).

### CSN electrostimulation attenuates inflammation independently of the vagus nerve

**Fig. 4:**
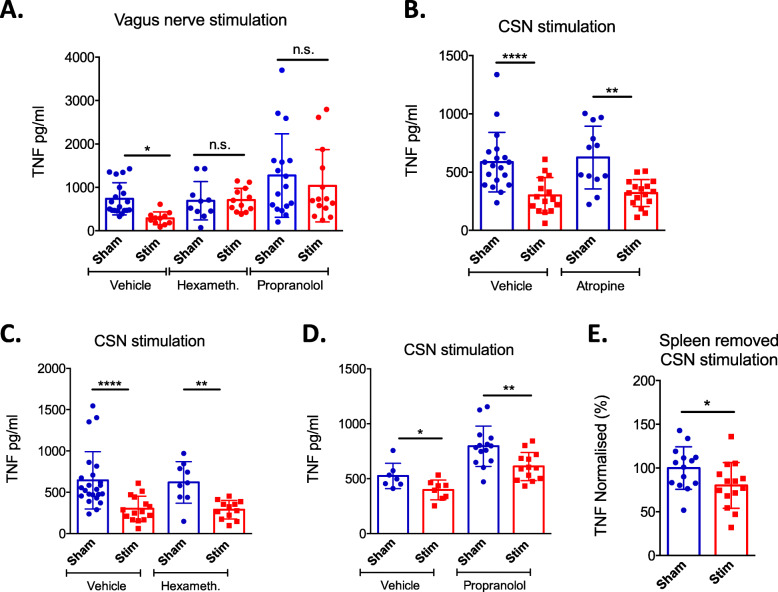
Attenuation of inflammation mediated via CSN stimulation does not utilise the vagal anti-inflammatory reflex. Eight to 10-week-old C57BL/6 mice were obtained, and vagus nerve (**a**) or CSN (**b**–**e**) isolation surgery was performed, followed by electrical stimulation 200–600 μA, 5–10 Hz. Thirty minutes prior to surgery, sham vehicle (PBS), hexamethonium (10 mg/kg) (**a**, **c**), atropine (1 mg/kg) (**b**) or propranolol (2.5 mg/kg) (**a**, **d**) were administered IP. Blood was collected 90 min after an IP LPS injection (100 μg) for serum analysis by ELISA. **e** The spleen was surgically removed, and the CSN electrostimulation was applied or not. LPS-induced serum TNF release was evaluated by ELISA. **a**–**e** Each individual point represents an animal, and data is expressed as means ± SD

Having shown that CSN electrical stimulation inhibited LPS-induced TNF production, we investigated the underlying mechanisms by using pharmacological antagonists. Since previous studies have shown that the inhibition of LPS-induced TNF secretion can be attenuated by signalling through acetylcholine receptor (AChR) and by β2 adrenergic receptor (AR) [4], we tested the impact of antagonism against these pathways examining CSN inhibition of LPS-induced cytokine release. To this aim, atropine, hexamethonium and propranolol which are respectively muscarinic, nicotinic AChR and β1/β2 AR antagonists were used (Fig. 1c for timeline). We first confirmed that these antagonists were effective at the doses used by showing that they prevented the decrease of LPS-induced TNF release following vagus nerve stimulation (Fig. 1d for timeline; Fig. 4a: *p* > 0.05, unpaired *t* test, *n* = 10–17). We then stimulated the CSN in the presence of atropine, hexamethonium and propranolol and found that the inhibition of LPS-induced TNF production was abolished neither by atropine (Fig. 4b: *p* = ***, unpaired *t* test, *n* = 12–16) nor by hexamethonium (Fig. 4c: *p* = **, unpaired *t* test, *n* = 9–12) nor by propranolol (Fig. 4d: *p* = **, unpaired *t* test, *n* = 13–14). LPS-induced TNF production was decreased by CSN stimulation after the surgical removal of the spleen (Fig. 1b for timeline; Fig. 4e: *p* = *, unpaired *t* test, *n* = 14). Overall, these results indicate that the effect of CSN stimulation is independent of the cholinergic anti-inflammatory pathway.

### The inhibitory effect of CSN stimulation on LPS-induced TNF production is dependent on the expression of GR by myeloid immune cells

**Fig. 5:**
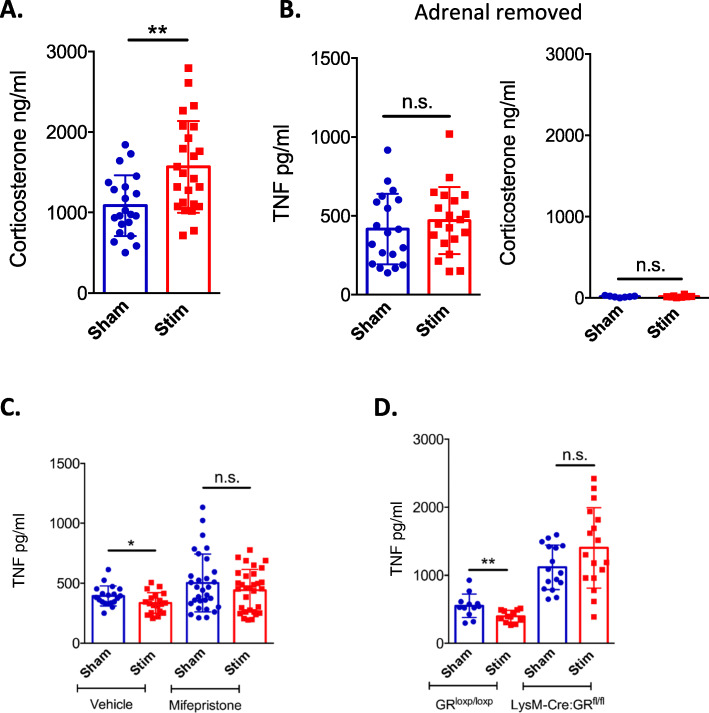
CSN electrostimulation attenuates inflammation via glucocorticoids signalling in myeloid cells. C57BL/6 mice (**a**–**c**) or LysM-Cre:GR^fl/fl^ mice (**d**) were obtained, aged 8–10 weeks, and CSN isolation and stimulation (600 μA, 10 Hz) were conducted. An LPS IP injection (100 μg) was administered in all animals; 90 min later, blood was collected for serum analysis by assay. **a** Corticosterone was measured in sham and CSN electrostimulated animals. **b** Impact of bilateral adrenal gland removal prior to CSN isolation and stimulation on LPS-induced serum TNF levels and serum corticosterone levels. **c** Impact of mifepristone (80 mg/kg) or vehicle (10% DMSO) IP administration prior to CSN isolation and stimulation on LPS-induced serum TNF levels. **d** LysM-Cre:GR^fl/fl^ mice and their littermate controls, GR^loxp/loxp^, were obtained, and both received electrostimulation of the CSN. LPS-induced serum TNF levels were measured by ELISA. All individual traces, or points, represent one animal. The means are represented as ± SD

**Fig. 6:**
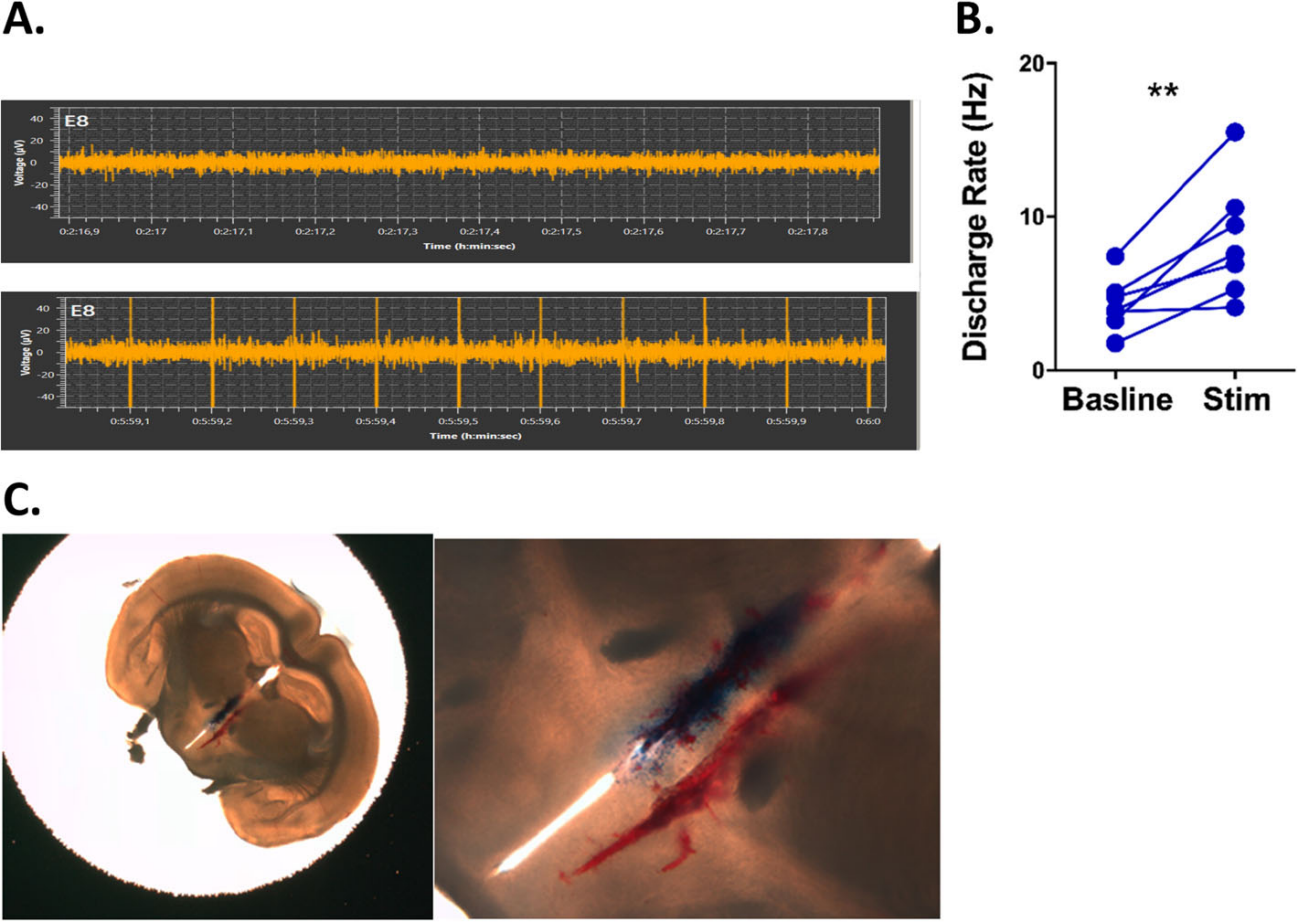
Increased PVN activity during CSN stimulation. Eight to 10-week-old C57BL/6 mice were obtained, and the CSN was isolated and stimulated (600 μA, 10 Hz). Electrical activity recordings of the PVN of the hypothalamus were taken using a stereotaxic frame. Impact of CSN electrostimulation on PVN activity was evaluated. **a** A representative trace of activity in the PVN during CSN stimulation. **b** The average discharge was acquired per minute for baseline recordings and during stimulation. All individual traces or points represent one animal. The means are represented as ± SD. **c** After recording, the frontal plane of a representative brain was injected with dye to show the location of the recording electrode and a zoomed in image of the PVN. Both images shown are representative of the whole

The HPA axis is a major neuroendocrine system that regulates immune response via the production of glucocorticoids by the cortex of the adrenal glands. To explore whether the inhibition of LPS-induced TNF production by CSN electrical stimulation could be dependent on the HPA axis, we applied electrical stimulation to the CSN and measured the serum levels of corticosterone. We found that CSN stimulation significantly increased the production of corticosterone (Fig. 1a for timeline; Fig. 5a: *p* = **, unpaired *t* test, *n* = 21–24). To investigate whether the ability of CSN electrical stimulation to inhibit LPS-induced TNF secretion was mediated by corticosterone, we bilaterally removed the adrenal gland in mice. While CSN electric stimulation did inhibit LPS-induced TNF secretion in sham-operated mice, adrenalectomy completely abolished this phenomenon (Fig. 1b for timeline; Fig. 5b, left: *p* = 0.4438, unpaired *t* test, *n* = 19–20). We confirmed that serum corticosterone levels were reduced by adrenalectomy (Fig. 1b for timeline; Fig. 5b, right: *p* = 0.9097, unpaired *t* test, *n* = 7–8). Since adrenal gland resection can affect other hormones than glucocorticoids, such as adrenaline, we administered mifepristone as a GR antagonist to mice. Treatment with the GR antagonist mifepristone also abolished the ability of CSN electric stimulation to inhibit LPS-induced TNF production (Fig. 1c for timeline; Fig. 5c: *p* = 0.4366, Mann-Whitney, *n* = 30–31). As myeloid cells, and more specifically macrophages, are the main source of TNF in LPS-injected mice, we investigated whether the inhibition of LPS-induced TNF production by CSN stimulation required the expression of GR by myeloid cells. To test this, we used LysM-Cre:GR^fl/fl^ mice in which myeloid cells are selectively deficient in GR. While CSN electrical stimulation did inhibit LPS-induced TNF production in GR^loxp/loxp^ littermates (Fig. 1a for timeline; Fig. 5d: *p* = **, unpaired *t* test, *n* = 13–14), it had no impact on LysM-Cre:GR^fl/fl^ mice (Fig. 5d: *p* = 0.0963, unpaired *t* test, *n* = 17–18) further demonstrating that GR signalling in myeloid cells was required for the inhibitory effect of CSN electrical stimulation. We further investigated the connection between the CB and the HPA axis by assessing the neural connection between the CSN and the PVN of the hypothalamus. We found that CSN stimulation did increase the activity in the PVN compared to baseline levels of activity (Fig. 1e for timeline; Fig. 6b: *p* = **, Mann-Whitney, *n* = 7). Altogether it is evident that CSN stimulation is activating the PVN which in-turn triggers the HPA axis and ultimately causes increased production of corticosterone, which inhibits LPS-induced inflammation by a GR-dependent and myeloid immune cell mechanism.

### CSN stimulation in conscious animals attenuates LPS-induced TNF production and increases survival to endotoxaemic shock

**Fig. 7:**
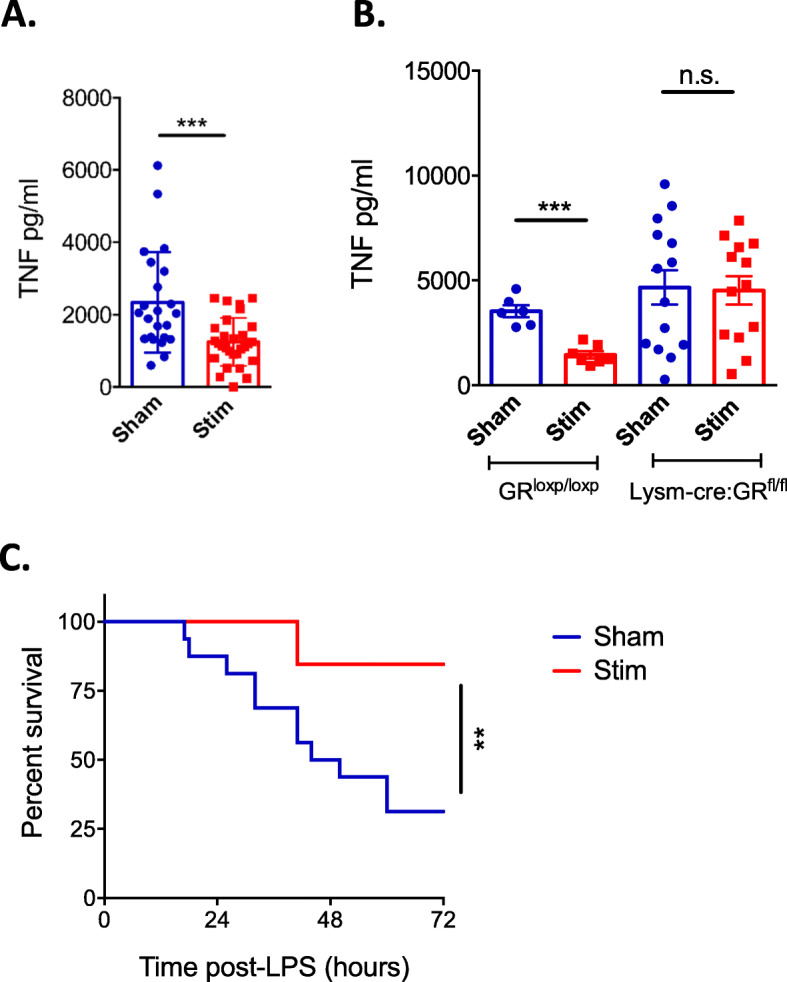
Conscious CSN stimulation is protective against LPS-induced shock. Eight to 12-week-old wild-type and transgenic C57BL/6 mice underwent surgery 4 days prior to stimulation (**a**–**c**). **a**, **b** Stimulation (200 μA, 5 Hz, 2 × 2 min) was applied on freely moving mice, and LPS (5 mg/kg) was injected IP. All individual points represent one animal, and the means are expressed as ± SD. **a** Impact of CSN electrostimulation in conscious animals on LPS-induced serum TNF levels. **b** LysM-Cre:GR^fl/fl^ mice and littermate controls underwent the same procedure as in **a**. **c** Wild-type C57BL/6 mice were implanted with an electrode on their CSN and stimulated (*n* = 16, 200 μA, 5 Hz, 5 min) or not (*n* = 13–16) twice a day for the next 3 days. A lethal dose of LPS (20 mg/kg) was administered to the mice (1 mg) IP. Animal survival was monitored

It is well documented that anaesthesia causes an anti-inflammatory effect [20]; therefore, it was necessary to confirm the previous results in fully conscious, non-anaesthetised animals. To this aim, we implanted electrodes onto the CSN and allowed the mice to recover. Mice were then injected with LPS, and electrical stimulation was applied. As observed in anaesthetised animals, CSN stimulation in conscious animals significantly decreased TNF concentration in the blood (Fig. 1f for timeline; Fig. 7a: *p* = ***, Mann-Whitney, *n* = 23–29). Furthermore, to replicate our results obtained in anaesthetised LysM-Cre:GR^fl/fl^ mice, LysM-Cre:GR^fl/fl^ mice and their littermate controls, GR^loxp/loxp^, were electrostimulated. Littermate controls had decreased TNF concentration following CSN electrostimulation (Fig. 1f for timeline; Fig. 7b: *p* = ***, unpaired *t* test, *n* = 6–7), whereas CSN-stimulated LysM-Cre:GR^fl/fl^ did not show a decrease in TNF concentration compared to sham-stimulated mice (Fig. 7b: *p* = 0.888, Mann-Whitney, *n* = 13–14).

We next investigated whether the inhibition of LPS-induced TNF production in conscious mice translates into increased survival following an endotoxaemic shock. We implanted electrodes underneath the CSN and allowed the mice to recover for several days. We then injected these mice with a lethal dose of LPS and applied electrical stimulation twice a day for 3 days. Conscious CSN stimulation of mice was protective against endotoxaemic shock-induced death (Fig. 1g for timeline; Fig. 7c: *p* = **, Gehan-Breslow-Wilcoxon test, *n* = 13–16).

Overall, our results suggest that CSN stimulation may prove to be a successful therapeutic for inflammatory disorders.

## Discussion

Here, we found that CSN electrical stimulation attenuates the production of pro-inflammatory cytokines via the increased production of corticosterone and a mechanism that is dependent on GR signalling in myeloid cells. This phenomenon was observed in both anaesthetised and non-anaesthetised conscious mice. Furthermore, CSN electrical stimulation translated into physiological benefit and protected mice from endotoxaemic shock-induced death, an observation that may be of clinical interest. Most importantly, the inhibition of LPS-induced TNF production by CSN electrical stimulation was not an artefact due to electrical spread to the vagus nerve. Interestingly, we also demonstrated that the stimulation is a purely afferent response.

It should be noted, however, that this afferent signal to decrease inflammation could potentially be mediated via the chemoreceptor or the baroreceptor sensors [8]. However, since the stimulation electrical parameters we used to decrease inflammation are sufficient to initiate the chemoreceptor response (Fig. 2a) but not the baroreflex response (Fig. 2b), it suggests that CSN electrostimulation is decreasing inflammation via a chemoreceptor reflex rather than the baroreceptor reflex. In line with these results, Brognara et al. showed that baroreflex stimulation did not impact serum, spleen and heart TNF levels following IP LPS injection [18].

In a recent paper, Santos-Almeida et al. demonstrated that CSN electrical stimulation in conscious rats attenuates inflammation and inhibits LPS-induced TNF production [17]. According to these authors, the inhibition of LPS-induced TNF production is dependent on signalling through both the AChR and the β1/β2 AR. They concluded that the CB is the afferent source of the vagal anti-inflammatory reflex as previously proposed [21]. In striking contrast, we found here that the inhibition of LPS-induced TNF production by CSN electrical stimulation in mice is neither abolished by the nAChR antagonist hexamethonium nor by the β1/β2 AR antagonist propranolol. These latter results led us to conclude that the mechanisms underlying the anti-inflammatory effect of CSN electrical stimulation are different from those involved in the vagal anti-inflammatory reflex. In line with this conclusion, the inhibition of LPS-induced TNF production by CSN electrical stimulation is not abolished by surgical removal of the spleen. Moreover, we also observed that the inhibition of LPS-induced cytokine secretion affects not only TNF and IL-6 but also IL-1 and IL-12p70 (Fig. 2a–d), which is not the case for vagus nerve stimulation (VNS) (personal communication). In conclusion, by contrast to VNS, the release of corticosteroids by CSN electrostimulation mediates its effects in a rather non-specific manner.

One possible explanation of the discrepancies between our results and those reported by Santos-Almeida et al. is that the physiological pathways that are mobilised by CSN electrical stimulation are different in mice and rats [17]. While this seems unlikely because of the phylogenetic proximity of these two species, it is noteworthy that fundamental differences in cytokine receptor profiles in CB have been reported between mice and rats [22–24]. Another explanation may be related to the differences in experimental procedures and more specifically to electrical stimulation parameters and regiments. In this respect, it is noteworthy that Santos-Almeida et al. have used a stronger than necessary signal and a protracted duration for activating the CSN [17]. We used amplitude of stimulation between 200 and 600 μA whereas Santos-Almeida et al. used 1-mA stimulation amplitude, which could lead to effects through the vagus nerve in addition to the CB-PVN. For example, this may have resulted in the artefactual recruitment of neural fibres in the vagus nerve and the subsequent triggering of the vagal anti-inflammatory reflex. We believe that this is a reasonable possibility as it was previously reported that a single electrical pulse is sufficient to activate the vagus nerve [25]. Furthermore, it is necessary to consider that their electrodes used were made of naked wires wrapped around the internal carotid artery, and this adds further likelihood of activating the close in proximity cervical vagus nerve. Regarding these points, we believe it is a reasonable possibility to suggest that there has been electrical spread to the vagus nerve instigating the vagal anti-inflammatory reflex. Unilateral removal of the vagus nerve might have confirmed that the attenuation of inflammation by rat CSN stimulation was independent of the vagus nerve. This control experiment would ensure that their effect of CSN stimulation did not occur due to electrical spread to the close in proximity vagus nerve, confirming or not the possibility of species differences.

Considering that CSN stimulation in mice is independent of the vagal anti-inflammatory reflex, we investigated other potential mechanisms for the effect of CSN stimulation. One possible mechanism for the attenuation of pro-inflammatory cytokines in mice by CSN electrostimulation is the HPA axis. There is evidence of a connection between the CB and the PVN of the hypothalamus—perhaps electrical activation of the CSN activates the PVN and instigates the HPA axis [26–28]. We first demonstrated that bilateral removal of the adrenal glands prevented the effect of CSN stimulation. Furthermore, we have shown that CSN electrostimulation is increasing serum corticosterone concentration. This result suggests that corticosterone is the ultimate mediator of the effect of CSN stimulation. This was confirmed by showing that the inhibition of LPS-induced TNF production by CSN electrical stimulation is abolished by treatment with the GR antagonist mifepristone or by inactivation of the GR signalling in myeloid cells. Overall, these results confirm that CSN stimulation acts via corticosterone activating GR on myeloid cells. It is known that there is a connection between the CB and the PVN [26–28]. The evidence, however, is effect-based or relying on c-fos studies. We confirmed the link between the CB and the PVN using an electrophysiological approach.

A method for conscious stimulation of the mouse CSN was developed, and it was demonstrated that conscious CSN stimulation can attenuate LPS-induced TNF production (Fig. 7a). Additionally, it was investigated if CSN stimulation in LysM-Cre:GR^fl/fl^ produced an effect. It was found that there was no difference between sham-stimulated and CSN-stimulated LysM-Cre:GR^fl/fl^ mice; this result indicates that the pathway for the effect of conscious CSN stimulation is concordant with that for anaesthetised stimulation.

We additionally investigated if this mitigation of inflammation by CSN electrostimulation could translate into physiological benefit—using a model of endotoxaemic shock by lethal LPS injection. To this aim, a method for conscious stimulation of the mouse CSN was developed, and it was demonstrated that conscious CSN stimulation can attenuate LPS-induced TNF production through the GR signalling in myeloid cells (Fig. 7b). We also found that CSN stimulation significantly increased the chance of survival in mice (Fig. 7c). This enhanced survival is likely to result from decreased production of pro-inflammatory cytokines such as TNF, IL-6, IL-12 and IL-1β, which play a critical role at recruiting and activating immune cells. For example, IL-12 activates natural killer (NK) cells while both IL-1β and IL-6 induce pyrogenic activity. TNF promotes the loosening of tight junctions between endothelial cells resulting in fluid loss and multiple organ failure [29]. Endotoxaemic shock is representative of immune system dysregulation, and as CSN stimulation can endow protection onto mice against it, it suggests that CSN stimulation may prove effective against additional IMIDs.

## Conclusion

CSN stimulation in mice activates the HPA axis enhancing the production of corticosterone, which in turn activates GR on myeloid immune cells ultimately mediating a decrease in inflammation, which aids survival in mice against endotoxaemic shock by lethal LPS injection. It may represent an interesting option in the anti-inflammatory bioelectronic medicine field as it is the first instance of an anti-inflammatory pathway using the HPA axis, which would be particularly interesting for patients requiring long-term administration of glucocorticoids.

## Data Availability

Not applicable.

## Abbreviations

ACh: Acetylcholine
AChR: Acetylcholine receptor
ACTH: Adrenocorticotrophic hormone
AR: Adrenergic receptor
CB: Carotid body
CRH: Cortisol-releasing hormone
CSN: Carotid sinus nerve
GR: Glucocorticoid receptor
HPA: Hypothalamic-pituitary adrenal
IBD: Inflammatory bowel disease
IL: Interleukin
IMID: Immune-mediated inflammatory disorders
IP: Intraperitoneal
LPS: Lipopolysaccharide
nAChR: Nicotinic acetylcholine receptor
NK: Natural killer
PVN: Paraventricular nucleus
RA: Rheumatoid arthritis
SD: Standard deviation
SLE: Systemic lupus erythematosus
TNF: Tumour necrosis factor
